# A Novel 3D Printed Model of Infected Human Hair Follicles to Demonstrate Targeted Delivery of Nanoantibiotics

**DOI:** 10.1021/acsbiomaterials.4c00570

**Published:** 2024-07-04

**Authors:** Samy Aliyazdi, Sarah Frisch, Tobias Neu, Barbara Veldung, Pankaj Karande, Ulrich F Schaefer, Brigitta Loretz, Thomas Vogt, Claus-Michael Lehr

**Affiliations:** † Department of Drug Delivery, Helmholtz Center for Infection Research, Helmholtz-Institute for Pharmaceutical Research Saarland, Campus E8 1, Saarbrücken 66123, Germany; ‡ Saarland University, Saarbrücken 66123, Germany; § Specialist in Plastic and Aesthetic Surgery, Saarbrücken 66111, Germany; ∥ Chemical and Biological Engineering, Rensselaer Polytechnic Institute, Troy, New York 12180, United States; ⊥ Clinic for Dermatology, University Clinic Homburg, Kirrberger Str., Homburg 66424, Germany

**Keywords:** *in vitro* model, hair follicle infection, follicular transport, 3D fabrication, tissue engineering

## Abstract

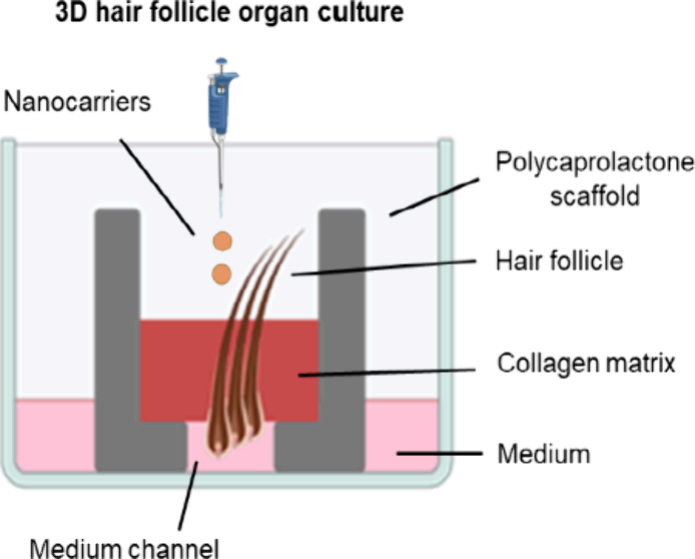

Hair follicle-penetrating nanoparticles offer a promising avenue for targeted antibiotic delivery, especially in challenging infections like acne inversa or folliculitis decalvans. However, demonstrating their efficacy with existing preclinical models remains difficult. This study presents an innovative approach using a 3D *in vitro* organ culture system with human hair follicles to investigate the hypothesis that antibiotic nanocarriers may reach bacteria within the follicular cleft more effectively than free drugs. Living human hair follicles were transplanted into a collagen matrix within a 3D printed polymer scaffold to replicate the follicle’s microenvironment. Hair growth kinetics over 7 days resembled those of simple floating cultures. In the 3D model, fluorescent nanoparticles exhibited some penetration into the follicle, not observed in floating cultures. *Staphylococcus aureus* bacteria displayed similar distribution profiles postinfection of follicles. While rifampicin-loaded lipid nanocapsules were as effective as free rifampicin in floating cultures, only nanoencapsulated rifampicin achieved the same reduction of CFU/mL in the 3D model. This underscores the hair follicle microenvironment’s critical role in limiting conventional antibiotic treatment efficacy. By mimicking this microenvironment, the 3D model demonstrates the advantage of topically administered nanocarriers for targeted antibiotic therapy against follicular infections.

## 1 Introduction

Hair follicles (HFs) are intricate structures comprising diverse cell types that fulfill vital roles in hair growth, cycling, and regeneration. Understandably, it is a complex task to mimic the physiological conditions *in vitro* for targeted drug testing. The various available models have specific applications and limitations. The pig ear model has been the most used for performing nanoparticle uptake studies as it has been established that nanosystems in the submicrometer size range can penetrate and accumulate within HFs.^1−4^ For instance, Lademann et al. showed how dye-loaded nanoparticles (320 nm) can effectively penetrate into HFs of pig ears.^5^ Another example by Raber et al. showed the uptake of poly(dl-lactide-*co*-glycolide) (PLGA) nanoparticles into pig ear HFs.^6^ Recently, follicular uptake was also shown for lipid-based nanoparticles on the pig ear model. While the pig ear model is suitable to demonstrate HF penetration due to high similarity,^7^ it is a tissue that cannot be cultured over a longer time. The lack of cultivability limits the use of biological efficacy and tissue or immune response. Additionally, the interspecies difference could lead to a distinct immune response. Human skin biopsies are another model for such nanocarrier penetration studies. Christmann et al. showed HF penetration of PLGA nanoparticles (150 nm) into skin biopsies of human body donors.^8^ Hair loss diseases, like alopecia areata, have been a major interest of such studies. However, once again, the lack of accessibility to intact HFs makes it difficult to analyze biological effects. Both models are suitable to be applied for demonstration of HF penetration with dye-loaded nanocarriers, but not for efficacy testing of drug-loaded systems. The presence of a functional HF holds the potential to unlock a multitude of applications, offering the possibility to assess the follicle’s condition in relation to various hair-related diseases.

Another approach uses isolated HFs cultured floating in a liquid medium. In 1990, Philpott et al. reported their HF organ culture from isolated human scalp skin, which was also applied for hair growth studies and hair loss diseases.^9^ This model’s strength is its suitability for biological effect investigation over several days. However, using such cultures for other applications, like drug delivery systems and bacterial infections, remains uninvestigated. A limitation arises from the fact that HFs, which are naturally enveloped by a three-dimensional matrix *in vivo*, cannot be effectively exposed to topical nanocarrier formulations or bacteria in this floating state.^9,10^ More recent methodologies incorporate tissue engineering, wherein dermal papilla cells are seeded within a 3D human skin construct to generate a complete HF model.^11^ While these models approximate *in vivo* conditions, they remain intricate, costly, and time-intensive.

Given these limitations, HF infection diseases have still not been approached *ex vivo* or *in vitro* for investigations. Acne inversa and folliculitis decalvans are two examples of such HF infections. Folliculitis decalvans manifests as an inflammatory scalp ailment, presenting lesions characterized by follicular pustules.^12,13^ In the pathogenesis of this disease, *Staphylococcus aureus* (*S. aureus*) emerges as a predominant microbe.^12,13^ Acne inversa, also termed hidradenitis suppurativa, represents another inflammatory disorder affecting the skin and HFs in regions like the axillary, genital, inframammary, or inguinal areas.^14^ Although the exact involvement of bacteria and the microbiome in the disease is still being investigated, *S. aureus* seems to play a role alongside other bacteria.^15^ Despite the clinical significance of such diseases,^16,17^ the mechanisms underpinning the treatment strategies remain inadequately comprehended. Typically, eradicating *S. aureus* is a primary objective aimed for but not always achieved through systemic or topical administration of antibiotics such as rifampicin.^18,19^ However, potential side effects of systemic approaches are a concern. In addition, antibiotic resistance and the development of biofilms by pathogens like *S. aureus* have been linked to treatment failures and the establishment of chronic conditions.^20,21^ To combat such infections, future approaches will necessitate novel anti-infectives delivered via advanced nanocarriers, acting as a reservoir at the site of infection. To study such anti-infective-loaded nanocarriers for topical administration, suitable disease models are required. The aforementioned approaches are not suitable for such studies. Considering the floating HF approach, which is already unsuitable for topical nanocarriers, the presence of bacteria in a liquid medium would result in rapid unphysiological bacterial growth. The human skin biopsies and the pig ear model are also not suitable due to the lack of access to the HFs and the missing method to determine the efficacy of anti-infectives against the bacteria inside the HF. Hence, we aimed to synergize approaches involving isolated human HFs and 3D matrices. We devised a 3D model encompassing a collagen matrix within a 3D printed polycaprolactone (PCL) scaffold, with isolated human HFs perpendicularly embedded/seeded. Hair growth kinetics were measured and compared to conventional floating follicle cultures. The application of fluorescent spheres (200 nm) on both approaches highlighted the pivotal role of the HFs’ surrounding environment. Additionally, we established an infection of the matrix-embedded HFs via *S. aureus*. To demonstrate the advantage of nanocarriers for targeted antibacterial drug delivery, we employed antibiotic-loaded lipid-based nanoparticles, which recently have shown promising results for follicular targeting on pig ears but also human skin biopsies.^22,23^ We prepared rifampicin-loaded lipid nanocapsules (LNCs) to compare their efficacy in both the infected 3D model and the conventional floating culture.

## 2 Materials and Methods

### 2.1 Bacterial Culture

*S. aureus* Newman GFP (ATCC 25904-pCtuf-gfp) was selected as a model pathogen to infect the HFs. For overnight culture, *S. aureus* was grown in 20 mL of a brain heart infusion medium with chloramphenicol, shaking (180 rpm) at 37 °C. Overnight culture was prepared by inoculating a colony from BHI-agar plates with chloramphenicol, streaked from glycerol stocks.

### 2.2 Hair Follicle Isolation

HFs were isolated from facial and abdominal skin tissues obtained from cosmetic surgery. Ethical approval (BU/170/20 Ethikkommission Ärztekammer des Saarlandes) and patient consent were obtained. The skin tissues were cut into 1–2 cm^2^ pieces and transferred to a Petri dish containing a washing buffer (phosphate-buffered saline (PBS), 5% fetal calf serum, 1000 U/mL penicillin, 1 mg/mL streptomycin, and 25 μg/mL amphotericin B, all from Life Technologies, UK). The epidermis and upper parts of the dermis were removed using a scalpel until the dermal-subcutis junction (grid pattern) was visible. HFs were gently pulled out under a stereomicroscope (Olympus, Germany) using fine tweezers. Anagen HFs, selected based on their morphology under the microscope, were collected in another Petri dish containing William’s E medium (Life Technologies, UK) with 500 U/mL penicillin, 0.5 mg/mL streptomycin, and 12.5 μg/mL amphotericin B. Subsequently, the HFs were transferred individually into single wells of a 24-well plate with 1.5 mL of fully supplemented William’s E medium (unless otherwise stated), with 10 μg/mL insulin (Sigma, Germany), 2 mM glutamine (Life Technologies, UK), and 10 ng/mL hydrocortisone (Sigma, Germany). The HFs were then incubated overnight before any experiments were performed.

### 2.3 3D Organ Culture of Hair Follicles

**Figure 1:**
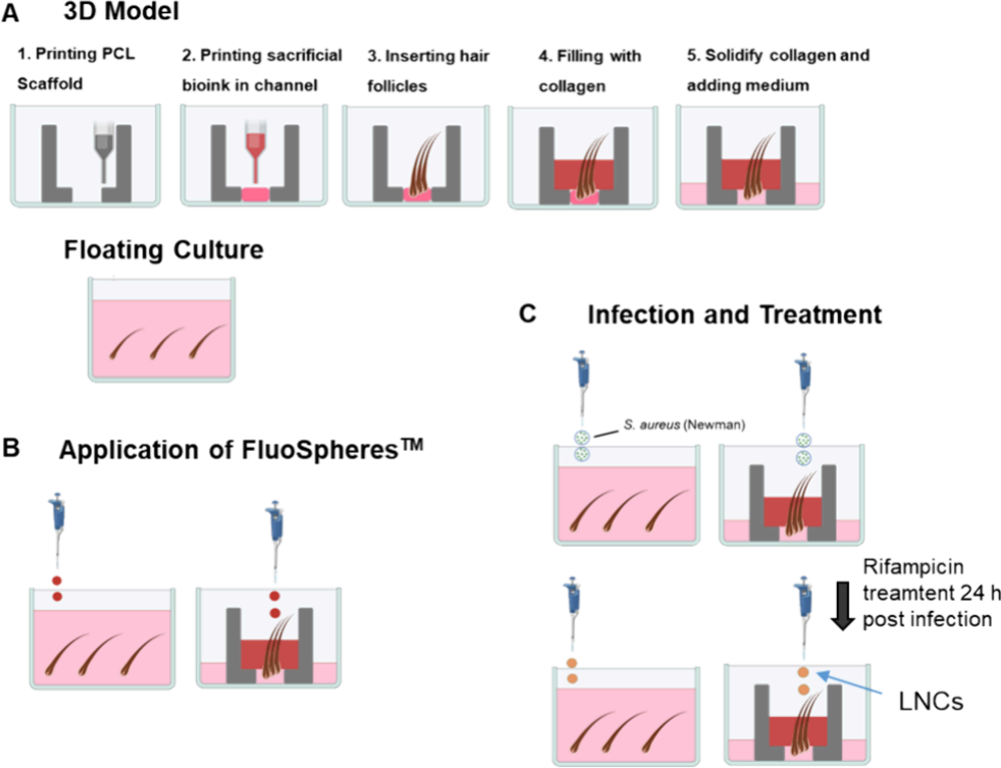
Scheme of procedures. (A) Scheme of the printing procedure for the 3D model and the HF insert. As control is the conventional floating culture of HFs. (B) Application of FluoSpheres for follicular transport on both culture types. (C) Infection of cultures via *S. aureus* Newman suspension drops with rifampicin treatment 24 h postinfection.

First, a PCL scaffold was 3D printed in a six-well plate, creating three channels that were subsequently filled by 3D printing a 25% Pluronic–F127 hydrogel (in William’s E medium and sterile-filtered before solidification) inside them (Figure 1A). The Pluronic serves as a sacrificial hydrogel to support the HFs to stay in position until the collagen is added. Accordingly, 1–3 HFs (specified for each experiment) were gently inserted into the tail (Corning, USA), composed of 80% (v/v) collagen, 10% (v/v) fully supplemented William's E medium, and 10% (v/v) neutralization solution (0.05 M NaOH, 2.2% NaHCO_3_, and 200 mM HEPES in Milli-Q), and were added into the PCL scaffold, ensuring that the HF tips remained in contact with air. The incorporation of collagen to this model intended to surround the HFs with an environment resembling the dermis, where collagen is the main extracellular component. The plate was then incubated for 10 min at 37 °C, before adding 1.5 mL (unless otherwise stated) of fully supplemented William's E medium to cover the bottom of the model, which also dissolved the Pluronic–F127 gradually. Additional technical details of the model are listed in Table S1.

### 2.4 Hair Growth Evaluation

Hair growth was assessed in the 3D model and the floating follicle approach. Due to limitations in the supply of skin tissues, only one HF was used in each culture. HFs were initially imaged under the stereomicroscope immediately after isolation. Only HFs that still exhibited the anagen phase after 1 day were used for the experiment. For the floating HFs, growth measurements were taken daily in the well plate. In the case of the 3D organ culture, each model was assigned to a specific day (with one HF per model) because the HFs were removed from the model for measurement under the stereomicroscope. The increase in hair length was evaluated using scale bar scale setting and line length measurement of ImageJ (NIH, USA).

### 2.5 Follicular Transport

To assess follicular transport, the floating culture and the 3D model were prepared as described previously. After 24 h, FluoSpheres carboxylate-modified microspheres (Thermo Fisher Scientific, Germany), red fluorescent spheres with a size of 200 nm, were diluted 1:10 in Milli-Q water. Then, 20 μL of the diluted solution was gently dropped on top of both cultures (Figure 1B). Follicular transport was assessed after 4 and 24 h. For this purpose, HFs were gently pulled using fine forceps and washed three times with 100 μL of PBS to remove all peripheral particles. Subsequently, the HFs were placed in a transparent 96-well plate (Black, Greiner Cellstar, Germany). Promptly, fluorescence imaging was performed using the live cell imaging system of a Spark Cyto (Tecan, Switzerland). Imaging was conducted with an LED intensity of 100% and an exposure time of 200 ms. Intensity profiles were generated by using the gray values of the received images. To do this, a region of interest was marked via ImageJ, covering only the HF, shown as a yellow rectangle. The intensity profile was then determined from the hair tip to the hair bulb, using the plot profile tool of ImageJ. To compare the floating culture with the 3D model, HFs of similar size were selected.

### 2.6 Hair Follicle Infection

To mimic an HF infection, the 3D models were infected with drops of *S. aureus* Newman (Figure 1C). The 3D model samples were prepared with three HFs. Accordingly, floating HFs were set up in a six-well plate with three follicles per well. An *S. aureus* overnight culture was adjusted to an OD_600_ of 0.2. Subsequently, 2 μL of the bacterial suspension was dropped onto either the 3D model, ensuring contact with the HF tips, or onto the floating approach, by pipetting the drop directly into the medium. The models were then incubated overnight at 37 °C.

### 2.7 Cytokine Release

We assessed cytokine release for interleukin 6 (IL-6), interleukin 8 (IL-8), and TNFα using a BD cytometric bead array (BD Biosciences, Germany). We prepared 3D HF models and infected them as described previously. The medium volume was reduced to 1 mL for this experiment to avoid excessive dilution. Noninfected models served as the control, as well as infected and noninfected floating HFs. We collected medium samples (60 μL) at 4 and 24 h after infection and stored them at −80 °C before performing the assay. The assay was conducted following the manufacturer’s protocol, using 50 μL of the samples for the measurements via a flow cytometer (BD LSRFortessa, BD Biosciences, Germany).

### 2.8 Rifampicin-Loaded LNCs

As a treatment option, LNCs were selectively loaded with rifampicin. These LNCs are composed of an oily core surrounded by layers of Span 80 and a PEGylated surfactant, based on the studies of Bastiat et al.^24^ To load LNCs with rifampicin, we employed a micromixing method. Briefly, we weighed 600 mg of soybean oil (Fisher Scientific, Germany), 600 mg of Migylol 812 (Caelo, Germany), 600 mg of Kolliphor (Sigma, Germany), 200 mg of Span 80 (Sigma, Germany), and 6 mg of rifampicin (US Biological, USA) into a vial and heated the mixture to 40 °C until the lipids melted. The contents were then transferred to a syringe (5 mL, Braun, Germany) compatible with the micromixer (IDEX, USA). Another syringe (10 mL) was filled with 8000 mg of Milli-Q water. For the Milli-Q, we set the flow rate to 2.320 mL/min, and for the oil mix, it was set to 0.580 mL/min. Flow rates were based on preliminary establishment, assuring no adverse effects on particle size or polydispersity. The diameter of the capillaries was 0.2 mm, ended in a 0.5 mm T-shaped micromixer. We discarded the droplets from the first 15 s and collected the remainder in another vial. The particle concentration was 200 mg/mL, calculated from proportions of flow rates. This was validated experimentally by freeze-drying samples and measuring the mass of the remaining lipid phases during preliminary establishment, which was consistent with the calculated mass.

### 2.9 Characterization of LNCs

#### 2.9.1 Dynamic Light Scattering

The size and PDI of the LNC formulation were investigated using dynamic light scattering. The measurements were carried out on a Malvern Zetasizer Nano ZSP (Malvern, Germany). The data were analyzed using Zetasizer software (Malvern, Germany). The measurements were performed with 12 different batches of the LNCs at 25 °C after 120 s equilibration in three repetitions, and then, the average value was determined. Before measurement, LNCs were diluted 1:400 in Milli-Q.

#### 2.9.2 Entrapment Efficiency and Drug Loading

The entrapment efficiency (EE) and drug loading (DL) were investigated using an indirect method. A 4% LNC suspension was filtered through an Amicon filter tube (Amicon Ultra-4, UFC810024, Merck Millipore, Ireland) with a 100.000 kDa molecular weight cutoff membrane. These filter tubes consisted of two chambers and a filter membrane. For that, 2 mL of the LNC suspension was placed in the upper chamber and filtered through the membrane at 150 g for 15 min (Rotina 420R, Hettich, Germany). Afterward, the concentration of rifampicin in the supernatant (lower chamber) was quantified using absorption measurement at 472 nm (Tecan Infinite 200 Pro, Tecan, Germany). To ensure that no LNCs were present in the supernatant, a dynamic light scattering measurement was performed. The EE and DL was then calculated using the following equations:
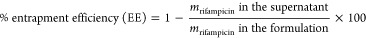

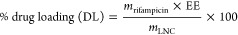


#### 2.9.3 Drug Release

Finally, the release behavior was also analyzed. For that, 2 mL of freshly rifampicin-loaded LNCs was transferred into dialysis tubes (MWCO: 3.5 kDa, Repligen, USA) and clipped on both sites. Tubes were transferred into 250 mL of Milli-Q. Releases were conducted over 24 h with gentle magnetic stirring. Samples (200 μL) were withdrawn after 1, 2, 4, 6, 8, and 24 h and pipetted into vials. Quantification of rifampicin was conducted on a Thermo Scientific Dionex UltiMate 3000 RS system with an Accucore RP-MS (Thermo Fisher Scientific, Germany) column. The mobile phase consisted of eluent A H_2_O with 0.1% (v/v) formic acid and eluent B acetonitrile with 0.1% (v/v) formic acid with a flow rate of 0.3 mL/min. The column temperature was set at 30 °C, while a 3 μL sample volume was injected. Data acquisition and analysis were performed by Xcalibur software. Mass spectrometry analysis was carried out on a TSQ Quantum Access MAX triple-quadrupole mass spectrometer system fitted with an electrospray interface operated under selected reaction monitoring transitions. The *m*/*z* transitions for rifampicin were 823.295 → 150.966 *m*/*z* and 823.295 → 162.927. Analyst Xcalibur software was used for instrument control and quantitative data analysis.

### 2.10 Treatment of Hair Follicle Cultures with LNCs

As a proof of concept, infected HF cultures were treated with rifampicin-loaded LNCs. To achieve this, 3D cultures and floating HFs were prepared and infected as described previously, here with three HFs per culture. After 24 h, the cultures were treated with 20 μL of 1:40 diluted rifampicin-loaded LNCs (5 mg/mL, with 15 μg/mL rifampicin in accordance with DL), which were added on top of each culture (Figure 1C). In accordance with a release study (Figure 6A), a rifampicin concentration of ∼10 μg/mL can be expected. As controls, other cultures were treated with plain LNCs (not loaded with rifampicin), as well as with 20 μL of free rifampicin (10 μg/mL). These treatments were conducted for 24 h at 37 °C. Subsequently, all three HFs of each culture approach were collected and individually transferred via sterile tweezers into 1 mL of PBS (1 HF in 1 mL) and vortexed at maximum speed for 15 min. To determine the colony-forming units per mL (CFU/mL), 20 μL of each sample was added to 180 μL of PBS in 96-well plates. Serial dilution was performed via 1:10 steps in PBS with an automatic multichannel pipet (Eppendorf, Germany). Three drops of 20 μL were then added on BHI-agar plates and incubated overnight at 30 °C. Then, the CFU was determined at the lowest possible dilution, where colonies could be clearly distinguished and counted accurately.

### 2.11 Statistics

All experiments were performed in independent biological triplicates, each with technical triplicates (*N* = 3; *n* = 9), unless otherwise stated. Significance was checked for cytokine release via *t-*tests and for the rifampicin-loaded LNC treatment via one-way ANOVA with Tukey’s multiple-comparison tests. Statistical significance was defined as *p < 0.05, **p < 0.01, and ***p < 0.001. Error bars indicate standard deviation.

## 3 Results

### 3.1 Culture of Floating Hair Follicles

**Figure 2:**
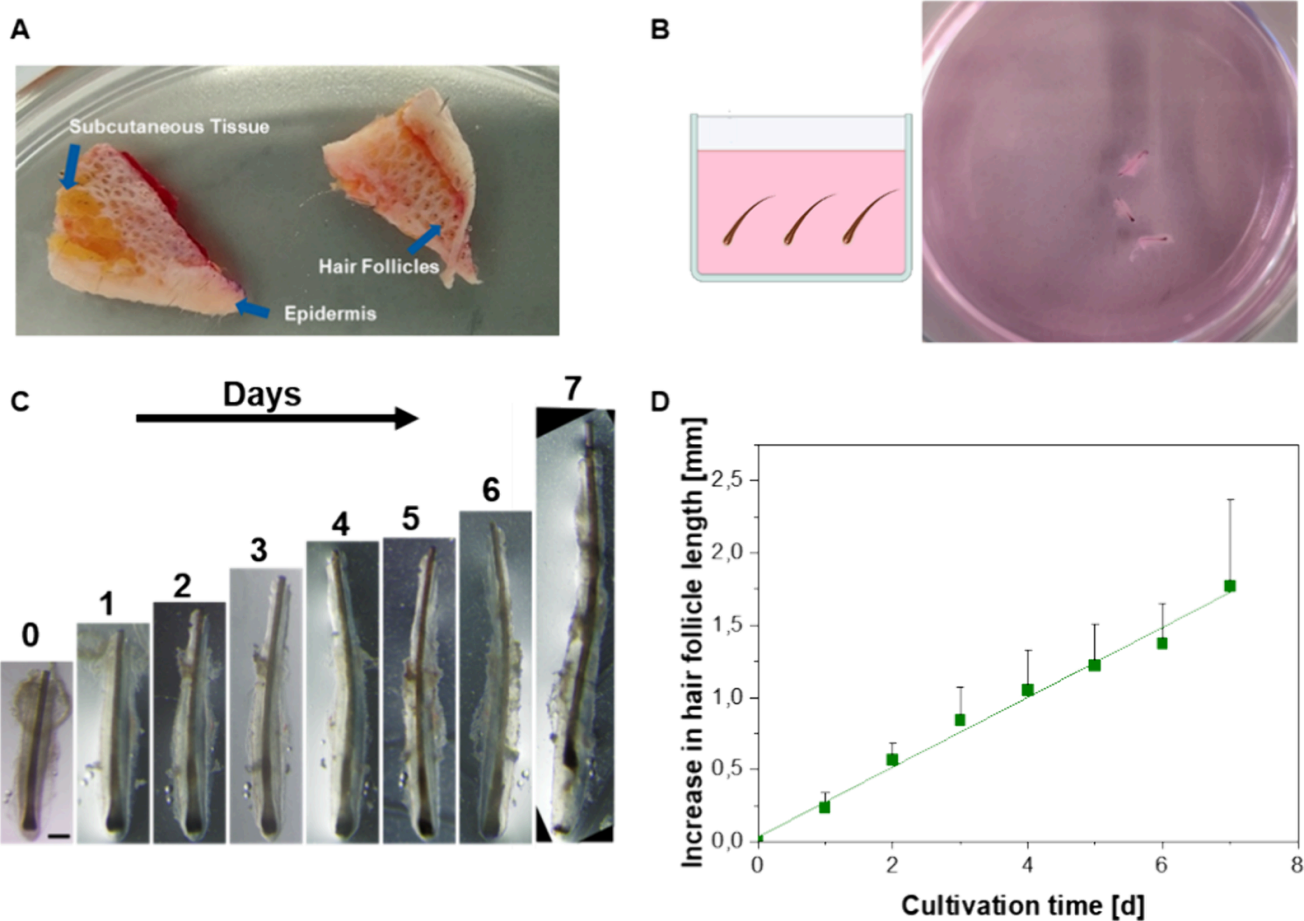
HF isolation and culture. (A) HF isolation from skin tissue via forceps. (B) Scheme and well of HF culture, floating within a medium. (C) Bright-field microscopy of a growing HF, cultured for a week. The scale bar equals 200 μm. (D) Quantification of HF growth over a week with *N* = 4; *n* = 12. Error bars represent standard deviation.

First, we isolated HFs from the skin tissue (Figure 2A) and assessed their cultivability by suspending them in a nutrient medium (Figure 2B). This was done to replicate a well-known cultivation method and provide a reference point for hair growth comparison. The HFs exhibited over 1 mm of growth within 7 days, maintaining a healthy anagen morphology until day 7 (Figure 2C,D). On approximately day 7, we observed a change in the HF cycle, as evidenced by the detachment of the hair fiber from the papillae.

### 3.2 3D Printed Hair Follicle Model

**Figure 3:**
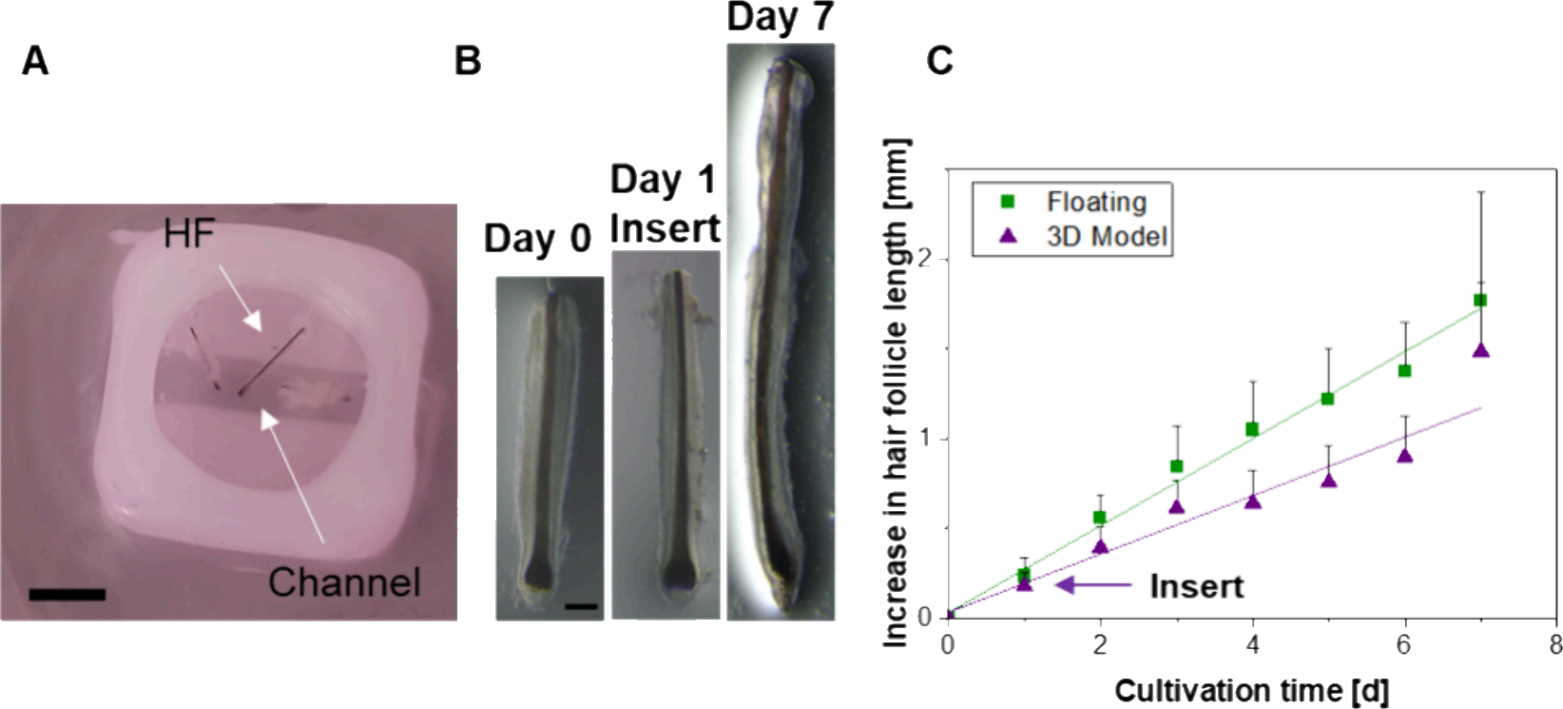
3D culture of HFs. (A) Image of the 3D model with 3 HFs. The scale bar equals 2 mm. (B) Bright-field microscopy of HF cultured from day 1 to 7 in the model. Images of other days depicted in Figure S1. The scale bar equals 200 μm. (C) Quantification of growth for both the 3D model and floating follicles with *N* = 2–4; *n* = 4–12.

To replicate the growth behavior within a 3D matrix, we designed a model that includes a PCL scaffold and a medium channel at the level of the HFs’ bulbs (Figures 1A and 3A). In preliminary experiments, we embedded HFs in collagen on commercially available Transwells but observed insufficient growth, probably due to poor nutrition supply. Therefore, we aimed to design a direct supply and optimized control via 3D printing. The increase of hair length in the 3D model closely resembled that of the floating HFs (Figure 3B,C). Due to limited availability of skin tissues, only one HF was used per model (instead of three). Daily growth images in the 3D model are provided in Figure S1. Hair fiber detachment occurred around day 7, indicating the success of the 3D culture within the surrounding matrix.

### 3.3 Follicular Transport

**Figure 4:**
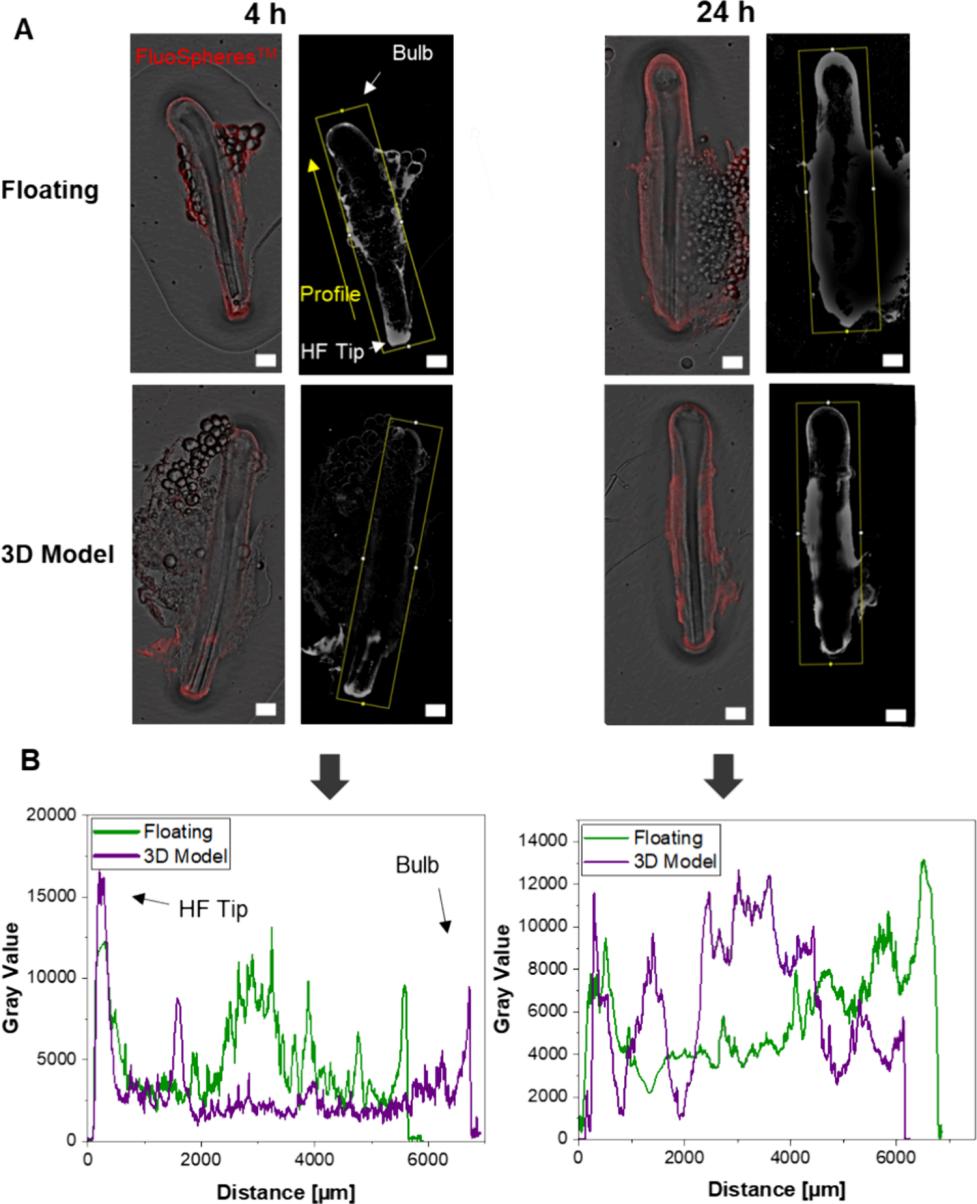
Follicular transport of FluoSpheres (200 nm). (A) Fluorescence imaging of spheres applied on top of the 3D model and floating HFs after 4 and 24 h (different HFs). The HF bulb is always upward and the HF tip is downward. The scale bars equal 100 μm. (B) Intensity profile of HFs after 4 and 24 h.

As described previously, nanocarriers in the submicrometer range show some accumulation in human HFs *in vivo*. Thus, we must postulate that this phenomenon should also be observed in a suitable *in vitro* model. For that, we assessed follicular transport using 200 nm fluorescent spheres. These spheres were applied to both the 3D model and floating HFs for 4 and 24 h (different HFs) (Figure 4A). Fluorescence imaging revealed that nanoparticles began to accumulate around the entire HF after just 4 h when the HFs were floating. In the 3D model, nanoparticles primarily gathered at the tip of the HF, suggesting that HF penetration occurs under such conditions, indeed, at least in most of the follicles. After 24 h, the 3D model exhibited deeper follicular penetration of nanoparticles, especially around the HFs, with some even detected inside the HF’s inner sheath as observed through fluorescence imaging. Additional replicates are displayed in Figure S2. Intensity profiles were generated from the gray value images, focusing on a region of interest within the yellow rectangle for HFs of similar size (Figure 4B). These profiles provide further insights, revealing a decrease in the gray value with an increasing distance from the hair tip in the 3D model, while the floating HFs exhibited a more consistent distribution over the entire follicle. After 24 h, the gray value remained higher over a longer distance before decreasing at the bulb level of the HF.

### 3.4 Hair Follicle Infection with *S. aureus*

**Figure 5:**
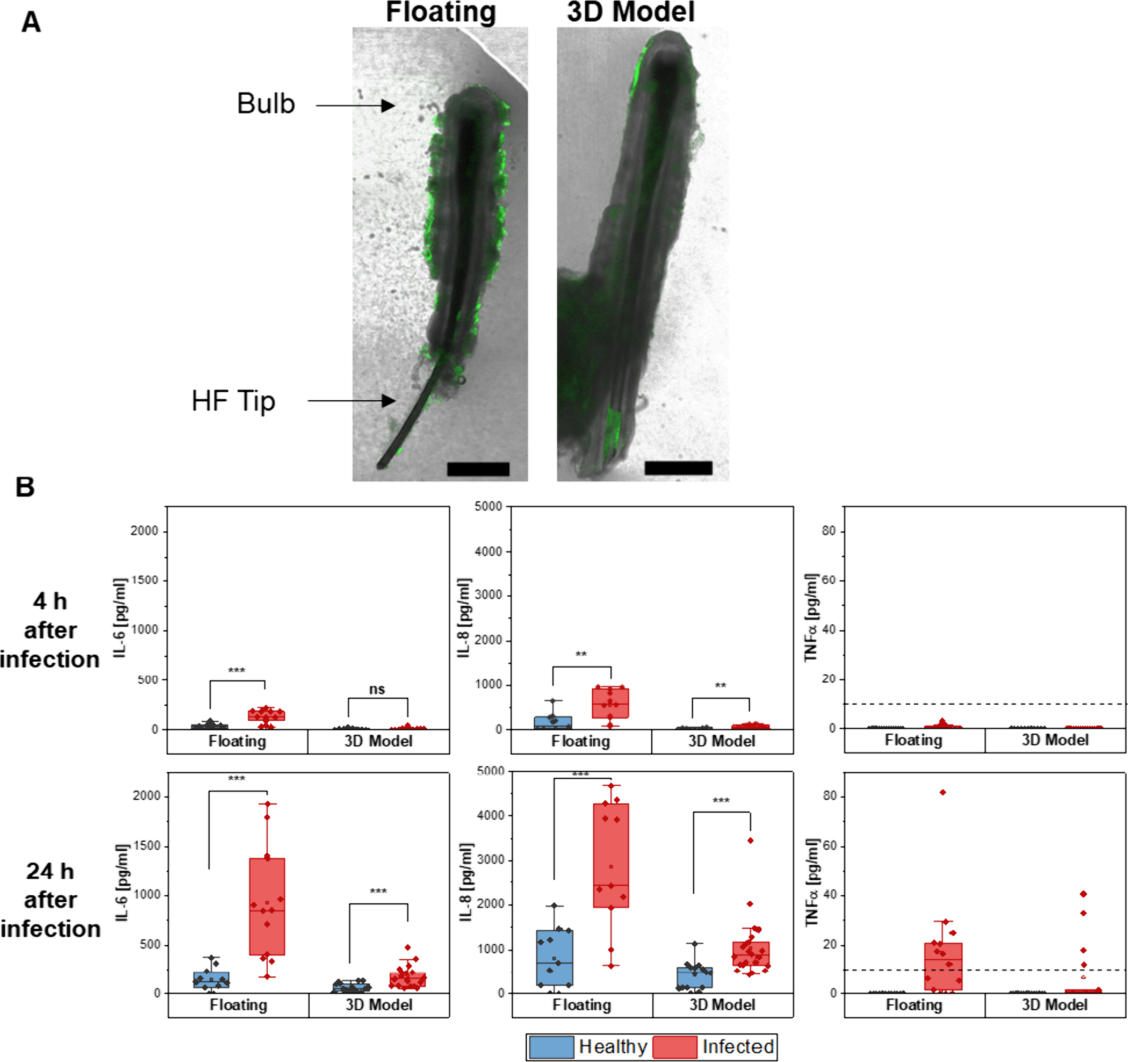
*S. aureus* infection of HF cultures. (A) Infection profile of HFs from both culture approaches via the GFP signal of bacteria. The scale bar equals 500 μm. (B) Cytokine release 2 and 4 h postinfection with *N* = 5–8 and *n* = 11–22 models (each with 3 HFs). Two-sample *t-*tests were performed for each condition; **p < 0.01, ***p < 0.001. Error bars indicate standard deviation.

To establish an infection model, we subjected the HFs to drop infection with *S. aureus* (Newman GFP). CLSM images taken after 24 h revealed that bacteria colonize the outer tissue surfaces, while some of the bacteria also appeared to enter the openings of the HF. This was, however, observed under floating conditions as well as in the 3D model (Figure 5A). As a measure of the HFs’ condition at 4 and 24 h postinfection, we assessed cytokine release, including IL-6, IL-8, and TNFα, using cytometric bead arrays in the surrounding medium. To determine the base level of cytokine release, noninfected HFs from the floating culture and the 3D model were also analyzed. After 4 h, a significant difference was already evident between the infected and noninfected conditions in the floating HFs, particularly for IL-6 and IL-8 (Figure 5B). In the 3D model, a slight but significant increase of IL-8 was observed. However, a baseline level of IL-6 and IL-8 release was already present in the noninfected conditions, indicating a stress response in the HFs, possibly due to the rigorous isolation procedure. At this time point, no TNFα was detected, likely due to the absence of immune cells postisolation. After 24 h, a strong, significant difference in IL-6 and IL-8 release was observed between the infected and noninfected HFs, suggesting that the bacteria induced an elevated cytokine release from the cells. Similar findings were observed in the 3D model, although with a lower overall release. Once again, TNFα was not significantly detected.

### 3.5 Rifampicin-Loaded Lipid Nanocapsules

**Figure 6:**
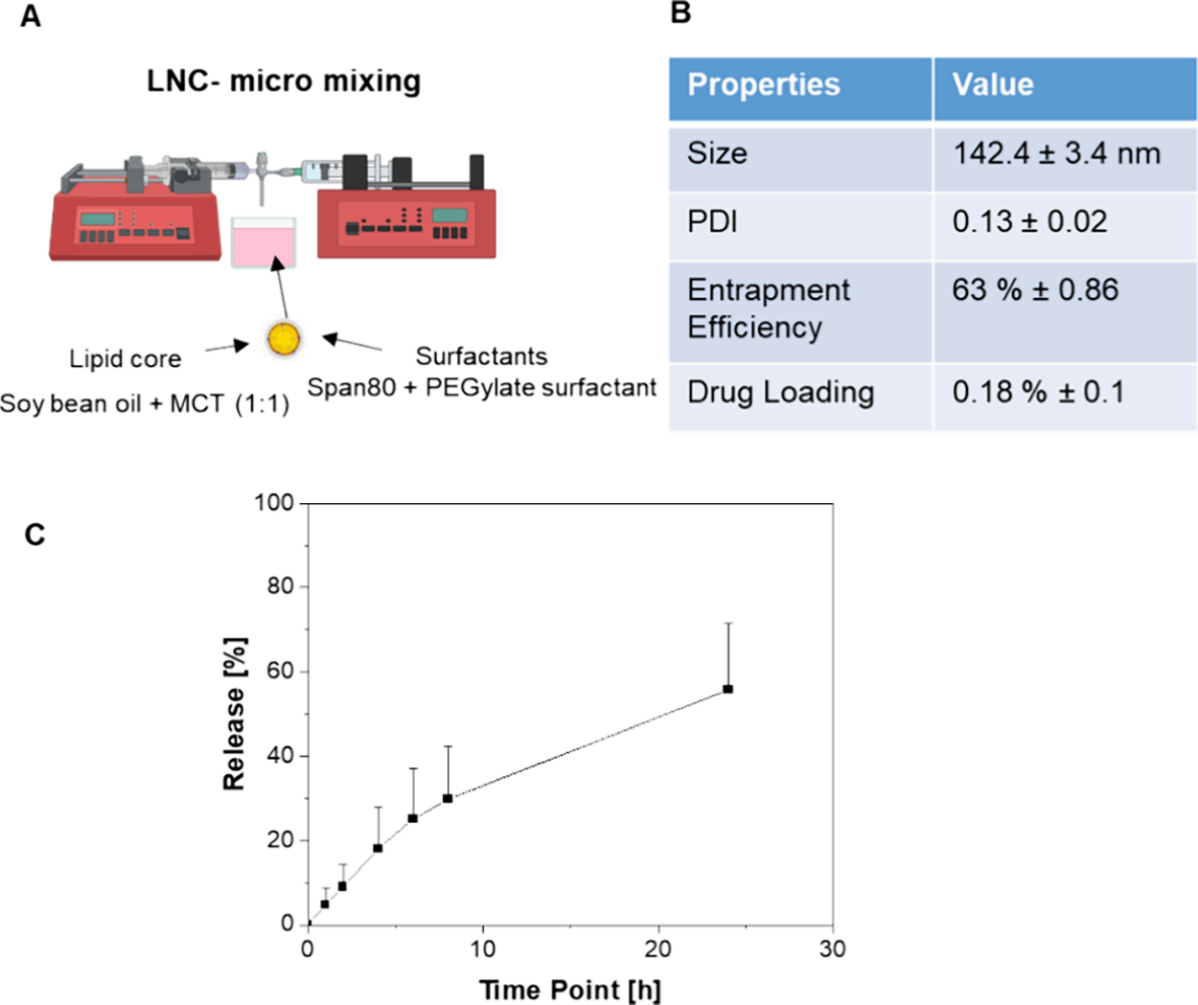
Characterization of LNCs. (A) Scheme of the micromixing process for LNC preparation. (B) Properties of LNCs with *n* = 12 for size and PDI and *n* = 3 for EE + DL. (C) Rifampicin release dynamics over 24 h, with *n* = 9.

LNCs were prepared via micromixing and subsequently characterized (Figure 6A). The LNCs exhibited a size of 142.4 ± 3.4 nm (Figure 6B), with a PDI of 0.13 ± 0.02. The determined EE was at 63% ± 0.86 and the DL at 0.18% ± 0.001. The release of rifampicin was about 60% of the drug load after 24 h (Figure 6C), corresponding to a release rate of 3–4 μg/mL. The working concentration of 5 mg/mL exhibited no cytotoxic effects on HaCaT cells (Figure S3). More details can be found in the Supporting Information. HaCaT cells were chosen as the cell line for this study due to the significant presence of keratinocytes in HFs.^25^

### 3.6 Treatment of Hair Follicles with Rifampicin LNCs

**Figure 7:**
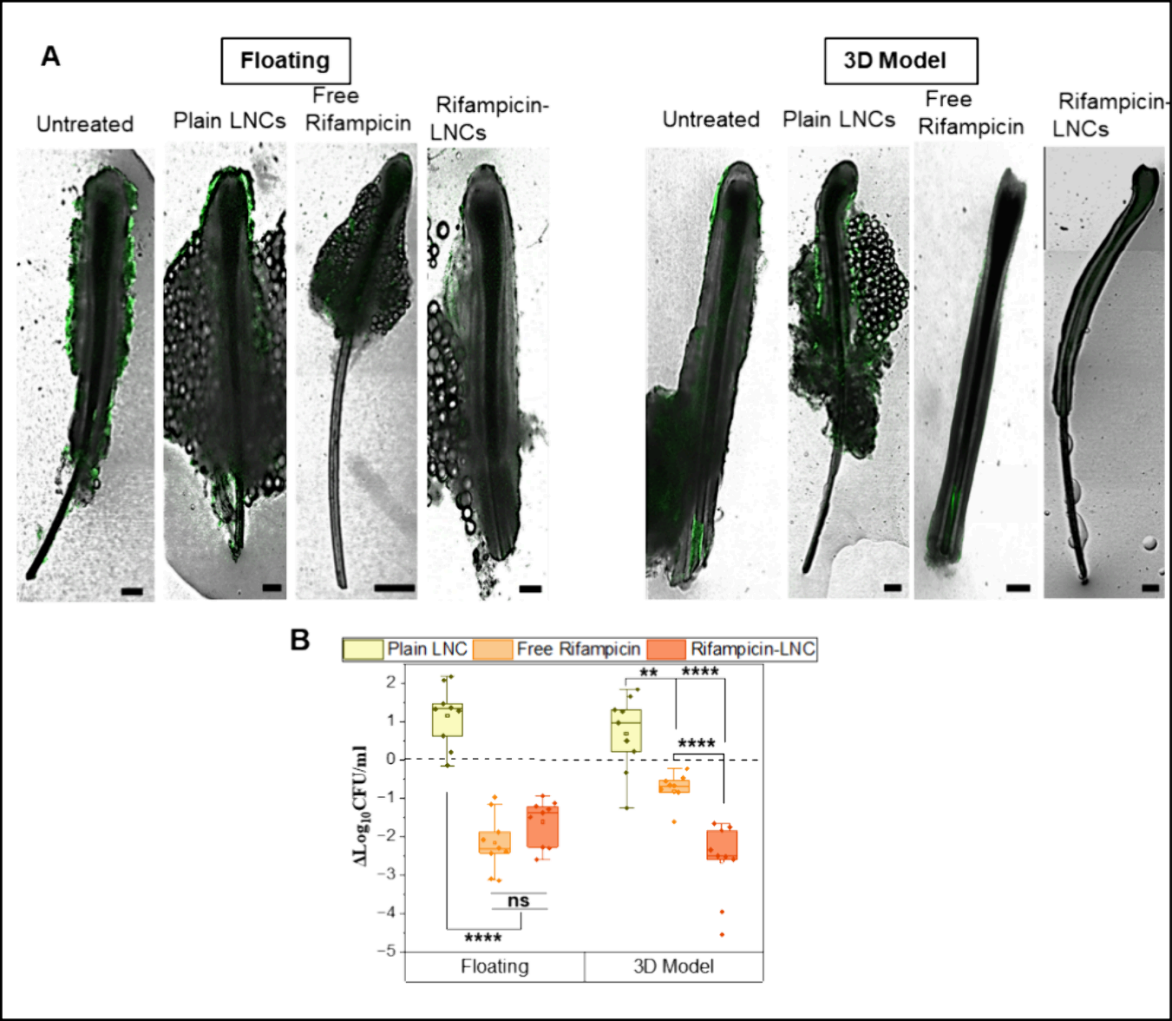
Rifampicin-loaded LNC treatment of infected HFs. (A) CLSM images of treated HFs via the GFP signal. Scale bars equal 200 μm. (B) Quantification of treatment via colony-forming units per mL of bacteria with *N* = 3; *n* = 9. Single-factor ANOVA with Tukey’s multiple comparisons was performed; **p < 0.01, ****p < 0.0001. Error bars indicate standard deviation.

The goal was to investigate whether the 3D model would be able to demonstrate a difference in antimicrobial activity when an antibiotic was delivered to the HF by some nanocarrier rather than in the free (i.e., molecularly dispersed) form. Therefore, we applied rifampicin-loaded LNCs to both the 3D and conventional culture infection models. Plain LNCs and free rifampicin (concentration adjusted to release from LNCs) served as control groups. Three HFs were used per 3D model, while analogous experiments were also conducted on HFs in conventional floating culture. CLSM images, showing the location and indicating the amount of GFP-expressing *S. aureus*, revealed no observable impact from the plain LNCs vs the untreated infection sample (Figure 7A). In the floating culture, a reduced GFP signal was observed when the free drug was administered, but no difference was observed when the rifampicin LNCs were used. A similar pattern was observed in the 3D model, but the images showed a slightly enhanced reduction in GFP when rifampicin was delivered by LNCs. To assess such differences in efficacy more quantitatively, the change in bacterial colony-forming units per milliliter (ΔCFU/mL) was determined (Figure 7B). As expected, plain LNCs had no discernible effect on the bacteria. In the floating culture, there was no significant difference observed between free rifampicin and rifampicin-loaded LNCs. Only in the 3D model, however, rifampicin-loaded LNCs demonstrated significantly stronger effectiveness compared to the free drug. These results suggest a crucial role of the 3D environment, obviously reflecting the follicular penetration of the nanocarrier, which cannot be observed with the free drug or with the conventional floating culture.

## 4 Discussion

Here, we report an innovative approach for culturing HFs within a 3D printed model to evaluate the effectiveness of nanoantibiotics against bacterial infections. Apart from a general lack of *in vitro* models addressing bacterial infections in HFs, the conventional HF cultures with floating follicles are not suitable for assessing the improved efficacy of anti-infective agents when delivered by nanocarriers. With our approach, we aimed to overcome these limitations. Initially, we conducted experiments using floating HFs to confirm the feasibility of culturing HFs after their isolation. When cultured over the course of 7 days *ex vivo*, the HFs exhibited a healthy anagen morphology. However, on the seventh day, we observed a change in the HF cycle, with the hair fibers detaching from the bulb. It is noteworthy that similar outcomes were previously documented by Philpott et al. in 1990, as well as by Khidhir et al. in 2013, and several other research groups.^9,26−28^ Subsequently, we cultured the HFs in a perpendicular orientation within a 3D printed model embedded in a collagen matrix. This approach yielded similar hair growth behavior, confirming the success of our method.

To verify the phenomenon of follicular penetration by nanocarriers in the model, we applied commercially available FluoSpheres to both culture approaches. After 4 h, the particles accumulated on the entire follicle in the floating culture, while they gathered specially on the follicle opening in the 3D model. Internal follicular transport was observed on images, but not every time. After 24 h, the particles accumulate deeper around the HF, penetrating also the inner sheath in the 3D model. While it has been shown that the optimal size for deep follicle penetration is between 400 and 700 nm, 100–200 nm exhibits also follicular penetration and quicker accumulation.^2^ Lademann et al. demonstrated that smaller particles perform more transfollicular transport, while optimal sized particles rather accumulate within the follicles.^5^ A remaining limitation of the model is here the missing possibility to massage the skin substitute. Moving the HFs by massage can cause a ratchet effect, enhancing nanoparticle penetration.^5,29−31^ Additionally, the HF isolation removed the upper part of the HF opening, removing an option for the particles to accumulate there as a reservoir. To solve that, HFs could possibly be isolated more gently and efficiently by using some dedicated follicular unit extraction (FUE) machines, which are also used for hair transplantations in the clinic^32^ and may keep the complete HF opening intact. This would help to assess nanoparticle penetration much closer to the *in vivo* situation.

Another crucial aspect of our study was infecting the model and observing the response of the HFs. To achieve this, we inoculated the cultures with bacteria and monitored the infection using fluorescence microscopy, specifically by tracking the GFP signal. Additionally, we analyzed the release of cytokines. Our observations revealed that the bacteria colonized the entire HFs in all cultures, including the surrounding area, the outer sheath, and the HF opening. Thus far, only a limited number of studies have visualized infections within HFs. In histological sections of skin biopsies with inflamed follicles, Jahns et al. demonstrated the colonization of *S. aureus* both inside and at the opening of the HF.^33^ Ten Broeke-Smits et al. confirmed that *S. aureus* has the capability to utilize HFs as colonization niches, both on the outer and inner portions of the follicles.^34^ Animal experiments involving mice produced similar results, with bacteria colonizing HFs deeply through the follicle openings.^35,36^ Following the infection, the HFs began to release increased amounts of cytokines, particularly IL-6 and IL-8, when compared to uninfected controls. This suggests that the HFs remain intact and respond to the infection. Keratinocytes and fibroblasts appear to be involved in this immune response.^37^ Notably, we did not detect the release of TNFα, likely due to the absence of immune cells. However, when comparing the floating cultures to the 3D model, we observed higher total cytokine levels in the former. This could be attributed to the different environments in which the cultures are exposed. In the case of floating HFs, they are in direct contact with bacteria to a greater extent than the follicles within the 3D model, where bacteria are applied on top. The collagen matrix within the 3D model serves as a barrier, potentially delaying bacterial colonization. However, there is no complete blocking between the collagen and the HF, allowing the bacteria also to colonize this area. Essentially, the 3D model provides a suitable readout via cytokine release when an infection is induced. Nevertheless, the model at this stage is limited by the lack of immune cell recruiting. Also, experiments may be performed to record the cytokine release after infection or inflammation and its treatment by therapeutic nanocarriers.

To evaluate our model in some specific application of follicular targeting, we designed rifampicin-loaded LNCs at a size of 142.4 nm, coming close to the size of applied FluoSpheres. Lipid-based nanosystems have already shown promising outcomes for follicle-targeted drug delivery applications on *ex vivo* human and porcine skin.^38−40^ Recently, Angelo et al. showed enhanced follicular deposition of lipid nanocarriers of similar size.^41^ While the EE and DL were not exceptionally high, they fall within an effective range. Essentially, the rifampicin release of 3–4 μg/mL after 24 h is above the minimum inhibitory concentration for *S. aureus*.^42^

As a final proof of concept, we treated the infected HF cultures with the well-known antibiotic rifampicin. In conventional floating cultures, there was no difference in efficacy between free rifampicin and rifampicin-loaded LNCs. However, in the 3D model, we observed a significant difference between the two treatments in the ΔLog10CFU/mL. The loaded LNCs appeared to be more efficient than the free drug, indeed, but were only observed in the 3D model and not on floating follicles. This difference can be attributed to the distinct environments in these two models. In the floating HFs, both the free drug and the drug-loaded LNCs can equally access the entire HF. In the 3D model, however, the LNCs have a better ability to enter the HF and penetrate the collagen, the same as it is known to occur *in vivo*, thus providing more efficient antibiotic drug delivery to the site of infection. Considering that *in vivo*, the HFs are not exposed to the drug in the same way as the floating follicles, our model obviously provides a more physiological and thus relevant approach.

Obviously, floating HF models are not very suitable for studying anti-infectives and in particular nanoantibiotics against bacterial infections, whereas the here described 3D model with implanted HFs allows to address some of these challenges and moreover allows to use human tissues. Nevertheless, this technique is not meant to entirely replace but to complement, e.g., the pig ear model, which is still suitable for visualizing and quantifying particle and drug accumulation in hair follicles. Considering the advantages of our method, this approach could be further optimized by using HFs with complete openings, possibly using “FUE machines” to better mimic the *in vivo* situation. Implementing an epidermis substitute with better mechanical properties, whether created artificially using polymers or through cellular methods, could further improve such a model by allowing also the application of massage. In the long term, increasing complexity by incorporating additional cell types such as fibroblasts and immune cells could make the model even more representative of the human situation. These aspects were already realized in modern skin equivalents.^43,44^ A complex model of this nature would provide valuable insights into nanoantibiotic targeting, HF viability, and pathogen behavior, ultimately improving the predictiveness of drug efficacy and safety assessments.

## 5 Conclusions

We have pioneered a 3D HF model, utilizing state-of-the-art 3D printing technology. By embedding isolated human HFs into a collagen matrix with a nutrient channel, our model successfully showed growth behavior comparable to simple floating HF cultures. Our study elucidates the critical role of the HF culture environment for follicular transport and underscores the potential of nanoparticulate formulations for targeted drug delivery. Importantly, the 3D HF model demonstrates susceptibility to infection and exhibits a cytokine response when exposed to *S. aureus*, highlighting its physiological relevance. With its innovative design and implications for advancing healthcare materials, our approach represents a promising platform for investigating nanoantibiotics against HF infections, offering significant potential for future therapeutic developments in this domain.

## Data Availability

The data that support the findings of this study are available upon reasonable request from the authors.

## Supporting Information Available

Additional experimental details including images of HF growth in a 3D model of each respective day (Figure S1), follicular transport (Figure S2), toxicity characterization of rifampicin-loaded nanocapsules on HaCaT cells via a PrestoBlue assay (Figure S3), and more technical details of the printed 3D model in Table S1 (PDF)

## Notes

Ethical approval (BU/170/20 Ethikkommission Ärztekammer des Saarlandes) and patient consent were obtained for all human tissues used in this study.

## Abbreviations

HF: hair follicle
PLGA: poly(dl-lactide-*co*-glycolide)
PCL: polycaprolactone
LNCs: lipid nanocapsules
PBS: phosphate-buffered saline
IL-6: interleukin 6
IL-8: interleukin 8
EE: entrapment efficiency
DL: drug loading
FUE: follicular unit extraction

## References

[ref1] Lademann J.; Richter H.; Schaefer U. F.; Blume-Peytavi U.; Teichmann A.; Otberg N.; Sterry W. Hair follicles - a long-term reservoir for drug delivery. Skin pharmacology and physiology 2006, 19 (4), 232–236. 10.1159/000093119. 16679826

[ref2] Gu Y.; Bian Q.; Zhou Y.; Huang Q.; Gao J. Hair follicle-targeting drug delivery strategies for the management of hair follicle-associated disorders. Asian journal of pharmaceutical sciences 2022, 17 (3), 333–352. 10.1016/j.ajps.2022.04.003. 35782323 PMC9237597

[ref3] Pelikh O.; Eckert R. W.; Pinnapireddy S. R.; Keck C. M. Hair follicle targeting with curcumin nanocrystals: Influence of the formulation properties on the penetration efficacy. Journal of controlled release: official journal of the Controlled Release Society 2021, 329, 598–613. 10.1016/j.jconrel.2020.09.053. 33011240

[ref4] Krishnan V.; Mitragotri S. Nanoparticles for topical drug delivery: Potential for skin cancer treatment. Advanced drug delivery reviews 2020, 153, 87–108. 10.1016/j.addr.2020.05.011. 32497707

[ref5] Lademann J.; Richter H.; Teichmann A.; Otberg N.; Blume-Peytavi U.; Luengo J.; Weiß B.; Schaefer U. F.; Lehr C. M.; Wepf R. Nanoparticles--an efficient carrier for drug delivery into the hair follicles. Eur. J. Pharm. Biopharm. 2007, 66 (2), 159–164. 10.1016/j.ejpb.2006.10.019. 17169540

[ref6] Raber A. S.; Mittal A.; Schäfer J.; Bakowsky U.; Reichrath J.; Vogt T.; Schaefer U. F.; Hansen S.; Lehr C.-M. Quantification of nanoparticle uptake into hair follicles in pig ear and human forearm. Journal of controlled release: official journal of the Controlled Release Society 2014, 179, 25–32. 10.1016/j.jconrel.2014.01.018. 24486055

[ref7] Zambrano A.; Klein A. L.; Patzelt A. Analysis of the morphometric parameters of pig ear hair follicles. Skin research and technology: official journal of International Society for Bioengineering and the Skin (ISBS) [and] International Society for Digital Imaging of Skin (ISDIS) [and] International Society for Skin Imaging (ISSI) 2021, 27 (5), 730–738. 10.1111/srt.13008. 33606308

[ref8] Christmann R.; Thomas C.; Jager N.; Raber A. S.; Loretz B.; Schaefer U. F.; Tschernig T.; Vogt T.; Lehr C.-M. Nanoparticle Targeting to Scalp Hair Follicles: New Perspectives for a Topical Therapy for Alopecia Areata. J. Invest. Dermatol. 2020, 140 (1), 243–246.e5. 10.1016/j.jid.2019.05.028. 31276676

[ref9] Philpott M. P.; Green M. R.; Kealey T. Human hair growth in vitro. J. Cell Sci. 1990, 97 (Pt 3), 463–471. 10.1242/jcs.97.3.463. 1705941

[ref10] Langan E. A.; Philpott M. P.; Kloepper J. E.; Paus R. Human hair follicle organ culture: theory, application and perspectives. Experimental dermatology 2015, 24 (12), 903–911. 10.1111/exd.12836. 26284830

[ref11] Abaci H. E.; Coffman A.; Doucet Y.; Chen J.; Jacków J.; Wang E.; Guo Z.; Shin J. U.; Jahoda C. A.; Christiano A. M. Tissue engineering of human hair follicles using a biomimetic developmental approach. Nat. Commun. 2018, 9 (1), 5301 10.1038/s41467-018-07579-y. 30546011 PMC6294003

[ref12] Otberg N.; Kang H.; Alzolibani A. A.; Shapiro J. Folliculitis decalvans. Dermatologic therapy 2008, 21 (4), 238–244. 10.1111/j.1529-8019.2008.00204.x. 18715292

[ref13] Alessandrini A.; Bruni F.; Piraccini B. M.; Starace M. Common causes of hair loss - clinical manifestations, trichoscopy and therapy. Journal of the European Academy of Dermatology and Venereology: JEADV 2021, 35 (3), 629–640. 10.1111/jdv.17079. 33290611

[ref14] Vinkel C.; Thomsen S. F. Hidradenitis Suppurativa: Causes, Features, and Current Treatments. J. Clin. aesthetic Dermatol. 2018, 11 (10), 17–23. PMC6239161 30519375

[ref15] Nikolakis G.; Liakou A. I.; Bonovas S.; Seltmann H.; Bonitsis N.; Join-Lambert O.; Wild T.; Karagiannidis I.; Zolke-Fischer S.; Langner K.; Zouboulis C. C. Bacterial Colonization in Hidradenitis Suppurativa/Acne Inversa: A Cross-sectional Study of 50 Patients and Review of the Literature. Acta dermato-venereologica 2017, 97 (4), 493–498. 10.2340/00015555-2591. 27882387

[ref16] Alotaibi H. M. Incidence, Risk Factors, and Prognosis of Hidradenitis Suppurativa Across the Globe: Insights from the Literature. Clinical, cosmetic and investigational dermatology 2023, 16, 545–552. 10.2147/CCID.S402453. 36891064 PMC9987236

[ref17] Vañó-Galván S.; Molina-Ruiz A. M.; Fernández-Crehuet P.; Rodrigues-Barata A. R.; Arias-Santiago S.; Serrano-Falcón C.; Martorell-Calatayud A.; Barco D.; Pérez B.; Serrano S.; Requena L.; Grimalt R.; Paoli J.; Jaén P.; Camacho F. M. Folliculitis decalvans: a multicentre review of 82 patients. Journal of the European Academy of Dermatology and Venereology: JEADV 2015, 29 (9), 1750–1757. 10.1111/jdv.12993. 25682915

[ref18] Sillani C.; Bin Z.; Ying Z.; Zeming C.; Jian Y.; Xingqi Z. Effective treatment of folliculitis decalvans using selected antimicrobial agents. International journal of trichology 2010, 2 (1), 20–23. 10.4103/0974-7753.66908. 21188019 PMC3002406

[ref19] Orenstein L. A. V.; Nguyen T. V.; Damiani G.; Sayed C.; Jemec G. B. E.; Hamzavi I. Medical and Surgical Management of Hidradenitis Suppurativa: A Review of International Treatment Guidelines and Implementation in General Dermatology Practice. Dermatology (Basel, Switzerland) 2020, 236 (5), 393–412. 10.1159/000507323. 32408306 PMC8177083

[ref20] Matard B.; Meylheuc T.; Briandet R.; Casin I.; Assouly P.; Cavelier-balloy B.; Reygagne P. First evidence of bacterial biofilms in the anaerobe part of scalp hair follicles: a pilot comparative study in folliculitis decalvans. Journal of the European Academy of Dermatology and Venereology: JEADV 2013, 27 (7), 853–860. 10.1111/j.1468-3083.2012.04591.x. 22779760

[ref21] Asfour L.; Trautt E.; Harries M. J. Folliculitis Decalvans in the Era of Antibiotic Resistance: Microbiology and Antibiotic Sensitivities in a Tertiary Hair Clinic. International journal of trichology 2020, 12 (4), 193–194. 10.4103/ijt.ijt_98_20. 33376291 PMC7759064

[ref22] Pereira M. N.; Tolentino S.; Pires F. Q.; Anjos J. L. V.; Alonso A.; Gratieri T.; Cunha-Filho M.; Gelfuso G. M. Nanostructured lipid carriers for hair follicle-targeted delivery of clindamycin and rifampicin to hidradenitis suppurativa treatment. Colloids and surfaces. B, Biointerfaces 2021, 197, 111448 10.1016/j.colsurfb.2020.111448. 33181388

[ref23] Caverzan J.; de Jesus M.; Durán N. Nanostructured Lipid Carriers Loaded with 17-α-Estradiol Accumulate into Hair Follicles. J. Braz. Chem. Soc. 2020, 31, 1345 10.21577/0103-5053.20200018.

[ref24] Bastiat G.; Pritz C. O.; Roider C.; Fouchet F.; Lignières E.; Jesacher A.; Glueckert R.; Ritsch-Marte M.; Schrott-Fischer A.; Saulnier P.; Benoit J.-P. A new tool to ensure the fluorescent dye labeling stability of nanocarriers: a real challenge for fluorescence imaging. Journal of controlled release: official journal of the Controlled Release Society 2013, 170 (3), 334–342. 10.1016/j.jconrel.2013.06.014. 23792117

[ref25] Kiani M. T.; Higgins C. A.; Almquist B. D. The Hair Follicle: An Underutilized Source of Cells and Materials for Regenerative Medicine. ACS biomaterials science & engineering 2018, 4 (4), 1193–1207. 10.1021/acsbiomaterials.7b00072. 29682604 PMC5905671

[ref26] Philpott M. P.; Sanders D. A.; Kealey T. WHOLE HAIR FOLLICLE CULTURE. Dermatologic Clinics 1999, 17 (2), 315–329. 10.1016/S0733-8635(05)70090-5. 9238319

[ref27] Khidhir K. G.; Woodward D. F.; Farjo N. P.; Farjo B. K.; Tang E. S.; Wang J. W.; Picksley S. M.; Randall V. A. The prostamide-related glaucoma therapy, bimatoprost, offers a novel approach for treating scalp alopecias. FASEB journal: official publication of the Federation of American Societies for Experimental Biology 2013, 27 (2), 557–567. 10.1096/fj.12-218156. 23104985 PMC3545535

[ref28] Kloepper J. E.; Sugawara K.; Al-Nuaimi Y.; Gáspár E.; van Beek N.; Paus R. Methods in hair research: how to objectively distinguish between anagen and catagen in human hair follicle organ culture. Experimental dermatology 2010, 19 (3), 305–312. 10.1111/j.1600-0625.2009.00939.x. 19725870

[ref29] Busch L.; Keziban Y.; Dähne L.; Keck C. M.; Meinke M. C.; Lademann J.; Patzelt A. The impact of skin massage frequency on the intrafollicular transport of silica nanoparticles: Validation of the ratchet effect on an ex vivo porcine skin model. European journal of pharmaceutics and biopharmaceutics: official journal of Arbeitsgemeinschaft fur Pharmazeutische Verfahrenstechnik e.V 2021, 158, 266–272. 10.1016/j.ejpb.2020.11.018. 33264667

[ref30] Radtke M.; Patzelt A.; Knorr F.; Lademann J.; Netz R. R. Ratchet effect for nanoparticle transport in hair follicles. European journal of pharmaceutics and biopharmaceutics: official journal of Arbeitsgemeinschaft fur Pharmazeutische Verfahrenstechnik e.V 2017, 116, 125–130. 10.1016/j.ejpb.2016.10.005. 27810473

[ref31] Robertson T. A.; Sanchez W. Y.; Roberts M. S. Are commercially available nanoparticles safe when applied to the skin?. Journal of biomedical nanotechnology 2010, 6 (5), 452–468. 10.1166/jbn.2010.1145. 21329041

[ref32] Dua A.; Dua K. Follicular unit extraction hair transplant. Journal of cutaneous and aesthetic surgery 2010, 3 (2), 76–81. 10.4103/0974-2077.69015. 21031064 PMC2956961

[ref33] Jahns A. C.; Lundskog B.; Berg J.; Jonsson R.; McDowell A.; Patrick S.; Golovleva I.; Palmer R. H.; Alexeyev O. A. Microbiology of folliculitis: a histological study of 39 cases. APMIS: acta pathologica, microbiologica, et immunologica Scandinavica 2014, 122 (1), 25–32. 10.1111/apm.12103. 23656553

[ref34] ten Broeke-Smits N. J. P.; Kummer J. A.; Bleys R. L. A. W.; Fluit A. C.; Boel C. H. E. Hair follicles as a niche of Staphylococcus aureus in the nose; is a more effective decolonisation strategy needed?. J. Hosp. Infect. 2010, 76 (3), 211–214. 10.1016/j.jhin.2010.07.011. 20864209

[ref35] Nakamura K.; Williams M. R.; Kwiecinski J. M.; Horswill A. R.; Gallo R. L. Staphylococcus aureus Enters Hair Follicles Using Triacylglycerol Lipases Preserved through the Genus Staphylococcus. Journal of investigative dermatology 2021, 141 (8), 2094–2097. 10.1016/j.jid.2021.02.009. 33705795 PMC8316282

[ref36] Onunkwo C. C.; Hahn B. L.; Sohnle P. G. Clearance of experimental cutaneous Staphylococcus aureus infections in mice. Archives of dermatological research 2010, 302 (5), 375–382. 10.1007/s00403-010-1030-y. 20130894 PMC2877165

[ref37] Rahmani W.; Sinha S.; Biernaskie J. Immune modulation of hair follicle regeneration. NPJ Regen. Med. 2020, 5, 9 10.1038/s41536-020-0095-2. 32411394 PMC7214459

[ref38] Friedrich R. B.; Kann B.; Coradini K.; Offerhaus H. L.; Beck R. C. R.; Windbergs M. Skin penetration behavior of lipid-core nanocapsules for simultaneous delivery of resveratrol and curcumin. European journal of pharmaceutical sciences: official journal of the European Federation for Pharmaceutical Sciences 2015, 78, 204–213. 10.1016/j.ejps.2015.07.018. 26215463

[ref39] Kalvodová A.; Zbytovská J. Lipid nanocapsules enhance the transdermal delivery of drugs regardless of their physico-chemical properties. International journal of pharmaceutics 2022, 628, 122264 10.1016/j.ijpharm.2022.122264. 36209979

[ref40] Vidlářová L.; Hanuš J.; Veselý M.; Ulbrich P.; Štěpánek F.; Zbytovská J. Effect of lipid nanoparticle formulations on skin delivery of a lipophilic substance. European journal of pharmaceutics and biopharmaceutics: official journal of Arbeitsgemeinschaft fur Pharmazeutische Verfahrenstechnik e.V 2016, 108, 289–296. 10.1016/j.ejpb.2016.07.016. 27449632

[ref41] Angelo T.; El-Sayed N.; Jurisic M.; Koenneke A.; Gelfuso G. M.; Cunha-Filho M.; Taveira S. F.; Lemor R.; Schneider M.; Gratieri T. Effect of physical stimuli on hair follicle deposition of clobetasol-loaded Lipid Nanocarriers. Sci. Rep. 2020, 10 (1), 176 10.1038/s41598-019-56760-w. 31932640 PMC6957495

[ref42] Yang H.; Xu S.; Huang K.; Xu X.; Hu F.; He C.; Shu W.; Wang Z.; Gong F.; Zhang C.; Liu Q. Anti-staphylococcus Antibiotics Interfere With the Transcription of Leucocidin ED Gene in Staphylococcus aureus Strain Newman. Frontiers in microbiology 2020, 11, 265 10.3389/fmicb.2020.00265. 32194524 PMC7066085

[ref43] Stanton D. N.; Ganguli-Indra G.; Indra A. K.; Karande P. Bioengineered Efficacy Models of Skin Disease: Advances in the Last 10 Years. Pharmaceutics 2022, 14 ( (2), ). DOI: 319 10.3390/pharmaceutics14020319. 35214050 PMC8877988

[ref44] Choi K. Y.; Ajiteru O.; Hong H.; Suh Y. J.; Sultan M. T.; Lee H.; Lee J. S.; Lee Y. J.; Lee O. J.; Kim S. H.; Park C. H. A digital light processing 3D-printed artificial skin model and full-thickness wound models using silk fibroin bioink. Acta biomaterialia 2023, 164, 159–174. 10.1016/j.actbio.2023.04.034. 37121370

