# Supplementary material for: A Novel 3D Printed Model of Infected Human Hair Follicles to Demonstrate Targeted Delivery of Nanoantibiotics
Source: ACS Biomater Sci Eng. 2024 Jul 4;10(8):4947–57. doi: 10.1021/acsbiomaterials.4c00570 (PMC11322910; doi:10.1021/acsbiomaterials.4c00570)
Supplement: Supplementary file 1 — ab4c00570_si_001.pdf [file ab4c00570_si_001.pdf]

Supporting Information

A Novel 3D-Printed Model of Infected Human Hair Follicles to Demonstrate Targeted Delivery of Nano-Antibiotics

‡*Samy Aliyazdi*[ab], ‡*Sarah Frisch*[ab], *Tobias Neu*[ab], *Barbara Veldung*[c], *Pankaj Karande*[d], *Ulrich F Schaefer*[b], *Brigitta Loretz*[a], *Thomas Vogt*[e], *Claus-Michael Lehr*[ab]*

[a]Department of Drug Delivery, Helmholtz Center for Infection Research, Helmholtz-Institute for Pharmaceutical Research Saarland, Campus E8 1, 66123 Saarbrücken

[b]Saarland University, 66123 Saarbrücken, Germany

[c]Specialist in Plastic and Aesthetic Surgery, 66111 Saarbrücken, Germany

[d]Chemical and Biological Engineering, Rensselaer Polytechnic Institute, Troy, NY 12180 USA

[e]University Clinic Homburg, Clinic for Dermatology, Kirrberger Str., 66424 Homburg, Germany

*E-mail: claus-michael.lehr@helmholtz-hips.de

2.3 3D Organ Culture of Hair Follicles

Table S1: Technical details of printed 3D model

Feature	Value
Diameter of model	8 mm
Height of model	~ 6.4 mm (respectively 32 layers of PCL)
Width of medium channel	~ 2 mm
Volume of collagen	70-80 µL
Height of collagen matrix	~ 2 mm
Distance between inserted HFs	Not fixed/variable

3.2 3D Printed Hair Follicle Model

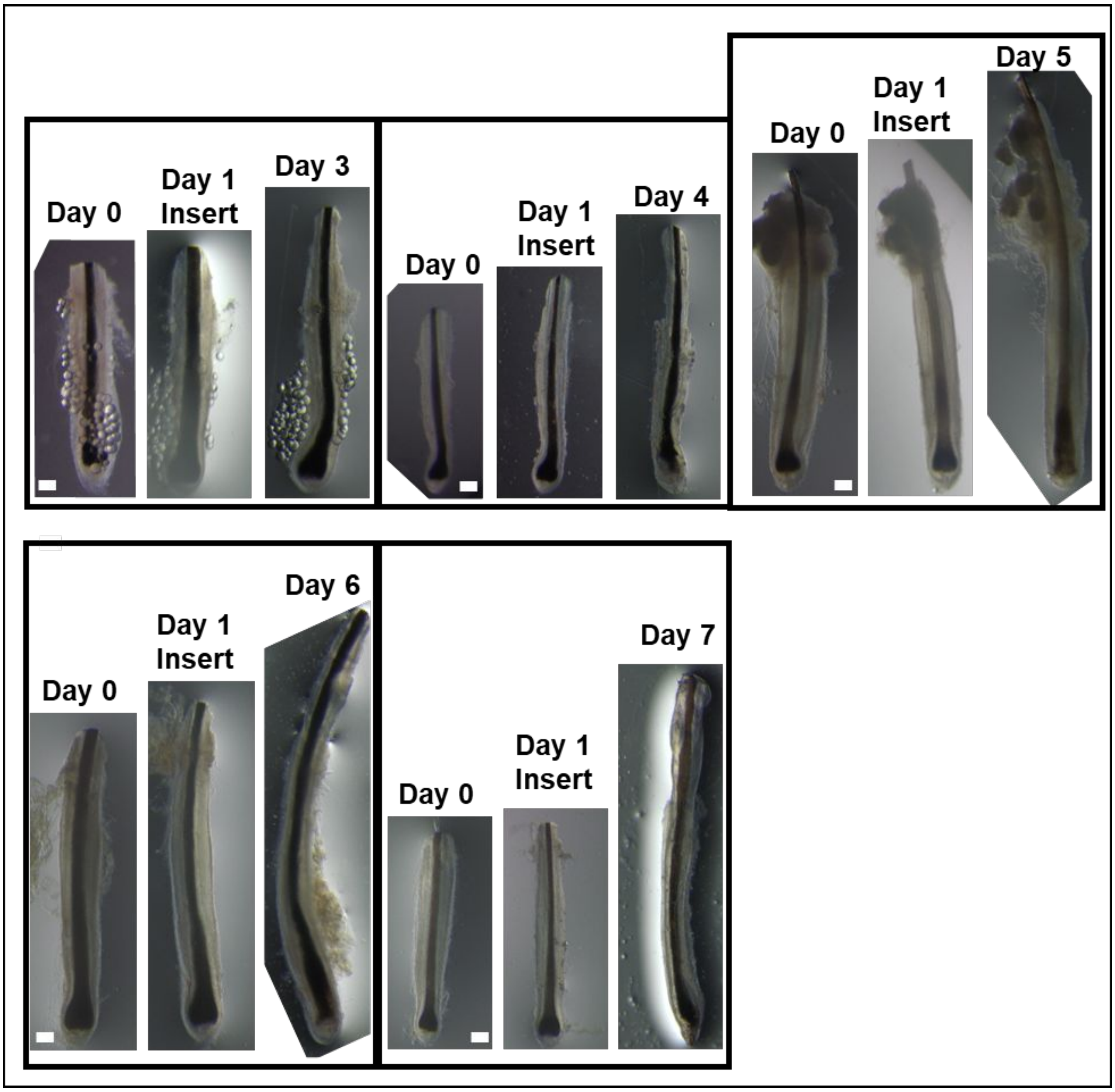

Figure S1: 3D culture of hair follicles. Images show growth of hair follicles in the 3D model from day 1 to 7. Scale bar equals 200 μm for every image.

3.3 Follicular Transport

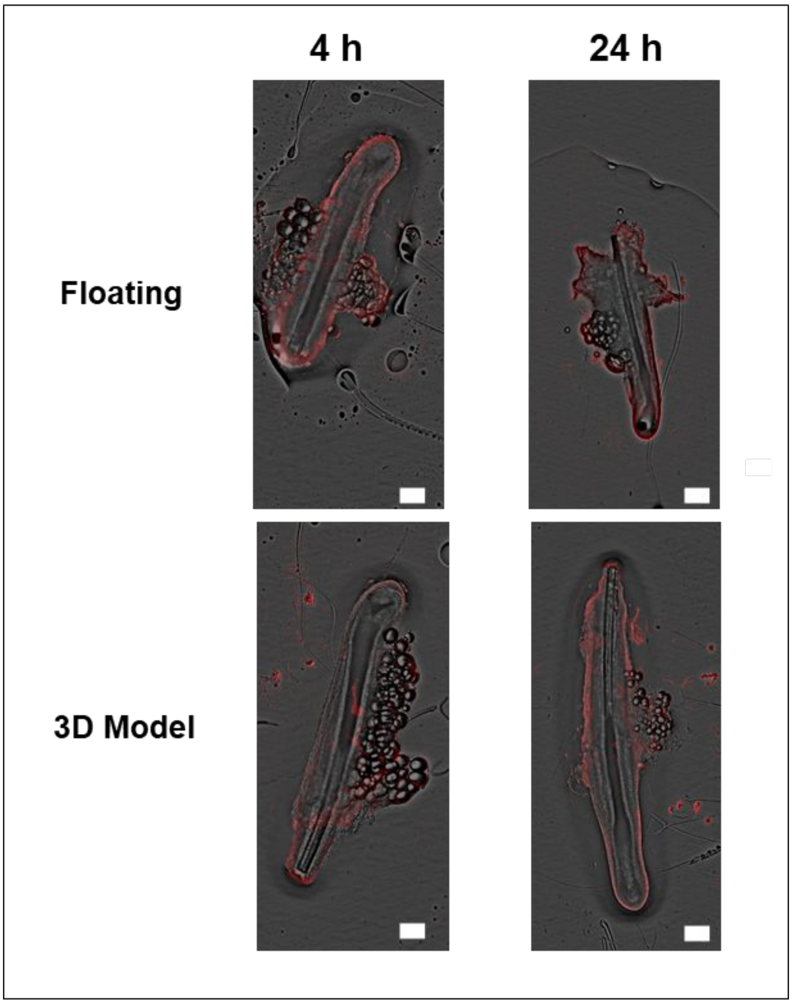

Figure S2: Follicular transport of Fluoroshperes (200nm). Additional replicates. Scale bars equal 100 µm.

3.5 Characterization of Rifampicin Loaded Lipid Nano Capsules

HaCaT cells were cultured in DMEM with 10% FCS at 37°C and 5% CO2. The cells were passaged once per week with medium change after 2-3 days. For testing the toxicity of rifampicin-loaded LNCs a Presto Blue assay was performed. For that, a 96 well plate was prepared with 200 µL DMEM + 10% FCS and 2*104 cells/well. After incubation for 3 days, medium was aspirated and 100 µL fresh medium was added to the wells. Treatment was performed using a 1:5 dilution of plain and rifampicin-loaded LNCs (or 80 µg/mL free Rifampicin) in PBS and for each, a 2-fold serial dilution was performed in PBS. 100 µL of the treatment was added to the wells with PBS serving as live and 1% (v/v) TritonX (Sigma-Aldrich, Germany) as dead control. Cells were incubated for 24 h before performing the assay. Subsequently, 10% (v/v) Presto Blue reagent was added to each well and the cells were incubated for an additional hour at 37°C and 5% CO2. Fluorescence intensity was measured at 560/590 Ex/Em using a plate reader (Tecan, Switzerland).

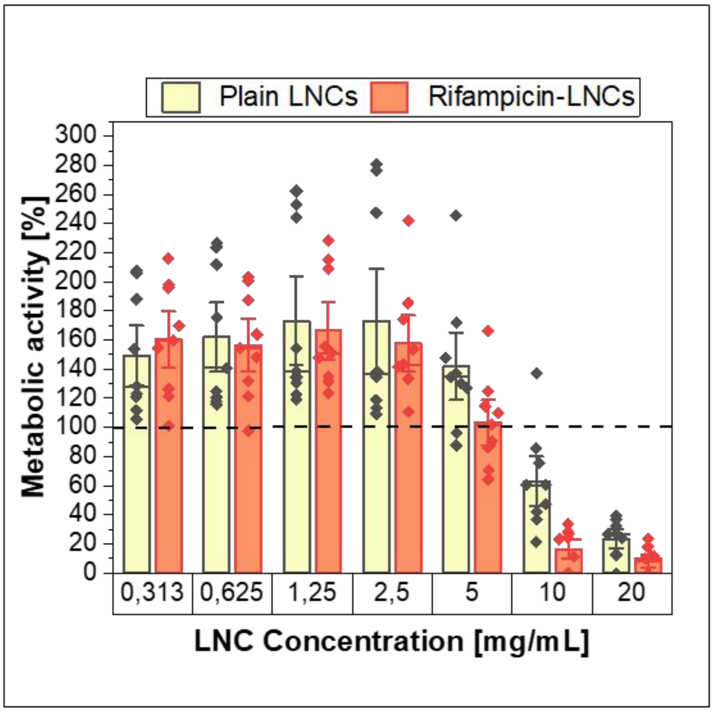

Figure S3: PrestoBlue assay with HaCaT cells to evaluate toxicity of LNC treatment. Error bars represent standard error with N=3, n=9.
